# Economic and social deprivation predicts impulsive choice in children

**DOI:** 10.1038/s41598-022-12872-4

**Published:** 2022-05-27

**Authors:** Richard J. Tunney

**Affiliations:** School of Psychology, Aston University, Birmingham, B4 7ET UK

**Keywords:** Psychology, Human behaviour

## Abstract

Impulsivity is an individual difference in decision-making that is a risk factor for a number of health concerns including addiction and obesity. Although impulsivity has a large heritable component, the health concerns associated with impulsivity are not uniformly distributed across society. For example, people from poorer backgrounds are more likely to be overweight, and be dependent on tobacco or alcohol. This suggests that the environmental component of impulsivity might be related to economic circumstances and the availability of resources. This paper provides evidence that children aged 4 to 12 from the most deprived areas in England show greater impulsivity in the form of delay discounting than do children from the least deprived areas. The data are discussed with reference to scarcity-based models of decision-making and to public health inequalities.

## Introduction

Why are some people more impulsive than others? The origins of most individual differences are little more than academic curiosity but impulsivity has a wide range of consequences for the individual and for society generally. Impulsivity is an individual difference that is defined by its behavioural consequences. For example, the overconsumption of food or alcohol is considered impulsive, as is gambling or shopping using expensive forms of debt such as credit cards. Similarly, unhealthy and potentially life-limiting behaviours such as tobacco use, or drug abuse, are also associated with impulsivity. On the other hand, life extending behaviours such as exercise, dietary restraint and abstinence from tobacco and alcohol are regarded as self-controlled, as is saving money for the future and living within one’s financial means. The relationship between impulsivity and mental health is not confined to addictive behaviours because impulsivity features as a diagnostic criterion in many seemingly unrelated disorders listed the Diagnostic and Statistical Manual of the American Psychiatric Association (DSM, and APA respectively) including Bipolar Disorder, Attention Deficit Hyperactivity Disorder (ADHD), and Antisocial Personality Disorder. The question of why some people are prone to making impulsive decisions is therefore of interest to the public health. This paper reports the results of a study that tested the hypothesis that environmental factors such as relative deprivation, is associated with impulsive choice in children.

Although the word impulsivity is used to describe a great many phenomena, it is typically characterised as a general time preference for smaller-sooner rewards instead of larger-later rewards^1^. Impulsive choice appears in early childhood. For example, in Mischel’s studies of delay of gratification^2^—the well-known marshmallow task—children are given a choice between a single marshmallow or, if they can resist temptation, two marshmallows sometime later. Video-recordings of children engaged in this task often show evidence that self-control is effortful for some children but not others. Observations of this sort led Mischel to hypothesise opponent hot (impulsive) and cold (controlled) processes^3^ that is reminiscent of other dual-process models of decision-making^4^.

A conceptually similar approach to the study of impulsivity the measurement of how much people discount delayed monetary rewards^5^. In a typical study, participants are given a series of choices between an immediate amount of money and a smaller amount of money to be received at some point in the future (e.g. would you prefer £10 now, or £20 in 12 months?). The delay period is titrated to determine the point at which the participant is indifferent between the immediate and the delayed outcome. This indifference point reveals the subjective discounted value of the value of the delayed reward. For example, if a decision maker was indifferent between the delayed reward of £25 in 12 months, and £10 immediately, we would say that the subjective value of the delayed £25 was discounted by £15. By titrating the delays and values of the outcomes it is possible to use the formula (1) to derive a single discount rate parameter (*k*)^6,7^ that numerically describes an individual’s relative preference for smaller-sooner or larger later rewards. Although the two measures are only imperfectly related to each other^8,9^ possibly because delay of gratification is behavioural and the delay discounting is psychophysical, or because they measure slightly different constructs. For the purposes of this paper the term time preference refers to any measure of impulsivity based on a preference for a smaller sooner reward or a larger later reward. Delay of gratification specifically refers to measures such as the Marshmallow Test developed by Walter Mischel. Delay discounting refers to psychophysical tests based on choices between monetary rewards that permit a discount parameter such as *k* to be derived. Some tests such as the one used in the English Longitudinal Study on Aging are based on choices between monetary rewards but do not permit *k* to be derived and are therefore referred to generically as a measure of time preference rather than discounting.

1 $$v=\frac{V}{1+kD}$$

Delay discounting is sufficiently associated with impulsive behaviours that discount rates can be considered a measure individual differences in impulsivity^10^, and as a significant risk factor for developing health problems. For example, daily smokers have higher discount rates than either social or non-smokers^11^, problem gamblers have higher discount rates than non-gamblers^12^, people who are dependent on opiates have higher discount rates than people who don’t use opiates^13^. Heavy drinkers discount the value of delayed rewards more steeply than light drinkers^14^, and people with larger Body Mass Indexes (BMI) have higher discount rates than people with healthy BMIs^15^. Discounting may even be a risk factor in the transition from recreational to problem gaming^16^, and is associated with symptoms of Conduct Disorder and ADHD^17^.

Individual differences in discounting emerge from 7 to 8 years old^9,18–20^ and remains stable throughout life suggesting that there may be a large heritable component^17,21^. Indeed, estimates of heritability suggest that around 50% of the variance in delay discounting and other measure of impulsivity are attributable to genetic factors^17,22–25^. This leaves around 50% of the variance attributable to environmental factors to be identified. Although it is already recognised that there are health inequalities across the UK relating to impulsive behaviour^26–28^, there is emerging evidence that the associated discount rates are not uniformly distributed across society and that these may be related to differences in economic circumstances. For example, Anokhin et al.^17^ noted that discount rates in 14 year olds were associated with socio-economic status based on parental occupation. More recently, Tunney and James^29^ reported that social-economic classification based on occupation predicted a measure of time preference similar to discounting in older adults. Why might a person’s occupation, or their parent’s occupation, predict how impulsive they are? Perhaps occupation is a proximal variable associated with a more fundamental driver of behavioural choice. One possibility is that a scarcity of resources or economic uncertainty leads the decision-maker to prefer immediate rewards when they become available, rather than waiting for larger and perhaps equally unpredictable rewards. In this respect, impulsive choice does not imply irrational choice. To test this hypothesis, Tunney and James^29^ compared preferences for smaller-sooner rewards in older adults from areas in England that ranged from the most deprived to the most affluent using the English Index of Multiple Deprivation (IMD). This is the official measure of relative deprivation in England and ranks each small area in terms of income, employment, education, health, crime, housing, and environment. Tunney and James observed that people in the most deprived areas were more likely to prefer smaller sooner rewards than people from the least deprived areas, and people in technical or routine occupations tended to prefer smaller sooner rewards than people in professional or intermediate occupations. Of course, the direction of a causal connection between working in a poorly paid profession and living in a deprived area, and impulsive choice cannot be established in older adults. It could be that impulsive people drift into poorly paid positions and towns based on the choices that they make. The study that follows seeks to test the hypothesis that that relative deprivation is related to impulsivity by measuring delay discounting in children aged between 4 and 12 years, from a range of backgrounds from the relatively deprived to the relatively affluent. If confirmed this would lend support to the hypothesis that deprivation is a causal environmental influence on impulsivity.

## Method

This study was conducted as part of the University of Nottingham Summer Scientist week in 2018. This is an annual event in which children from the Nottingham community take part in a wide range of psychological studies during the school holiday period.

### Participants

Informed consent to take part in this research was obtained from the parents or legal guardians of each participant. One-hundred and fifty-six children took part in this study. Their average age was 8.395 years (SD = 2.067). The youngest was 4.15 years old, and the oldest was 12.13 years old. Sixty-nine were female and 87 were male. The parents or legal guardians completed demographic information including post-codes that were used to derive the Index of Multiple Deprivation for their address, and also about ethnicity and the languages spoken in the home. The majority (143) indicated that English was the only language spoken at home, 2 Arabic and English, 2 Arabic, 3 English and Urdu, 1 French and English, 1 Japanese and German, 2 Mandarin, 2 did not respond to this question. All of the children were students in the British education system and were proficient in English.

### Ethics statement

The study was approved by the University of Nottingham School of Psychology Ethics committee and performed in accordance with the World Medical Association Declaration of Helsinki for ethical principles for medical research involving human subjects^30^ and the British Psychological Society’s Code of Ethics and Conduct^31^.

### Procedure

**Figure 1:**
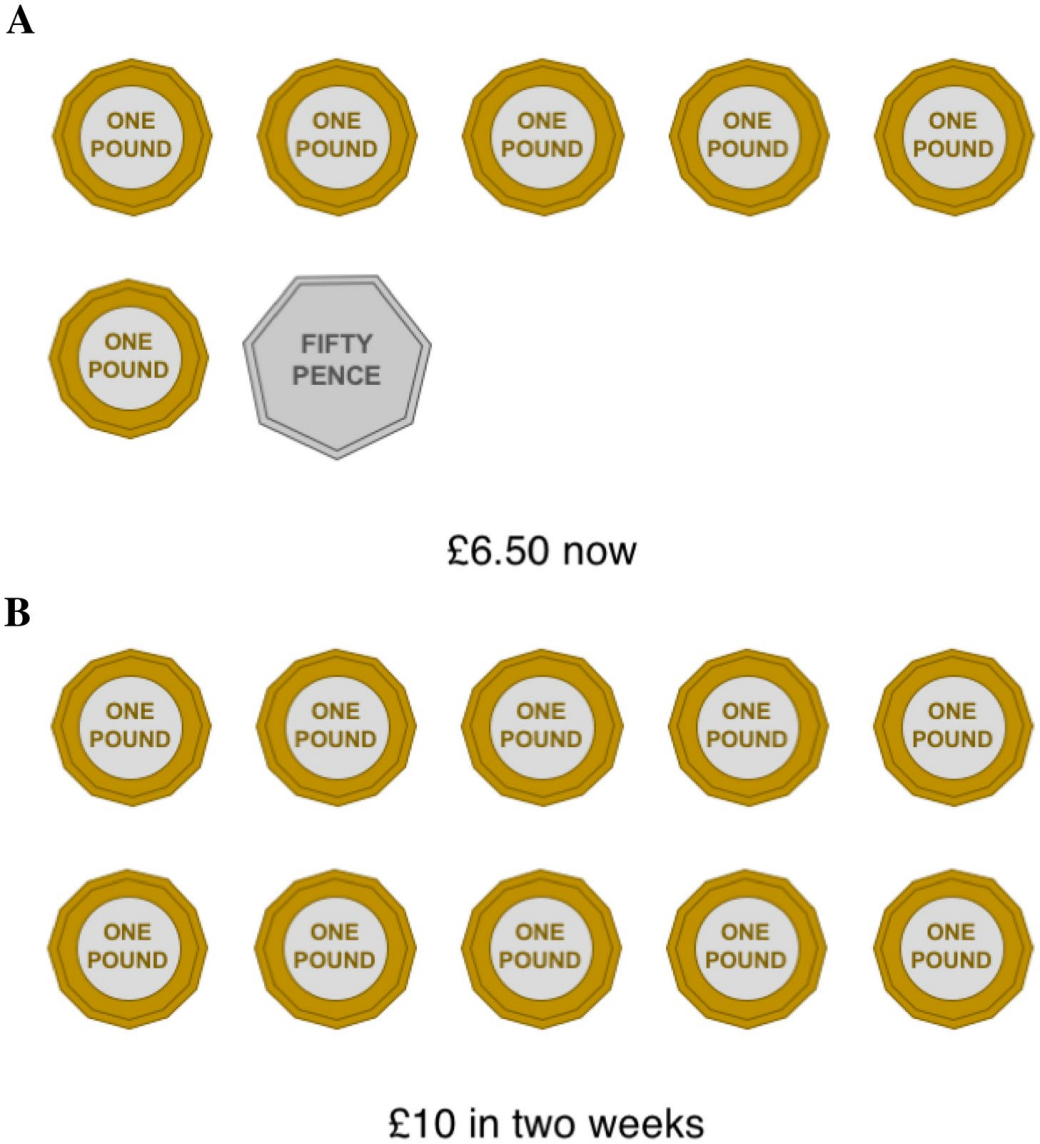
(**A**) Example stimulus depicting a smaller sooner reward. The value varied and the delay was fixed. Image created using Microsoft PowerPoint 2016. (**B**) Example stimulus depicting a larger-later reward. The value was fixed and the delay varied. Image created using Microsoft PowerPoint 2016.

There were five-time preference questions from which the parameter *k* was derived. On each trial the participants were given a choice between £10 after an interval or an immediate outcome that titrated upwards from £0.50 to £9.50 in 50p increments. These choices were displayed on an smaller-sooner card (Fig. 1A) and a larger later card (Fig. 1B) that showed the value of the choice in coins. The intervals were “in one-week” (coded as 7-days), “in two weeks” (14-days), “when school starts (30-days)”, “£10 at Christmas” (180-days), or £10 next summer” (365-days). For example, the child would first be asked “would you prefer £10 in one-week, or £0.50 now?” followed by “would you prefer £10 in one-week, or £1.00 now?”. The larger later card was titrated from the largest to the smallest value. Each trial would stop when the participant switched their preference from the larger later outcome to the smaller sooner outcome.

The participants completed the British Picture Vocabulary Scale III (BPVS-3)^32^ . The BPVS-3 is used to test receptive vocabulary in children ages from 3 to 18 years that provides an age adjusted standard score with a mean of 100. They also completed the Autism Quotient Children’s Version (AQ-Child)^33^ . The minimum AQ-Child score (0) indicates no autistic traits; the maximum score (150) suggests full endorsement on all autistic items. Finally, participants completed the Strengths and Weaknesses of Attention Deficit Hyperactivity Disorder Symptoms and Normal Behaviour (SWAN)^34^. This measure has two subscales for Inattention and Hyperactivity/ Impulsivity. Each test was age appropriate and the participants confirmed that they were able to comprehend instructions for each of the tests.

#### Results

**Figure 2:**
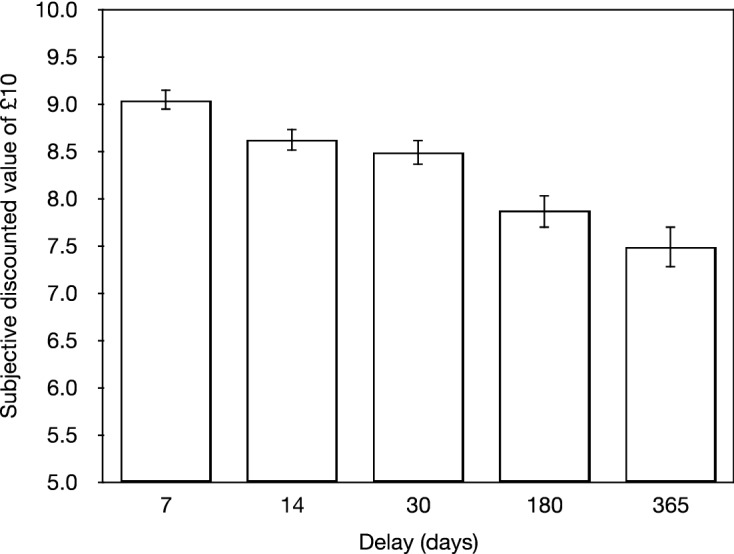
Showing the average subjective discounted value for each delay period. Error bars are standard errors of the mean.

Figure 2 shows the average subjective discounted value for each delay period. A repeated measures ANOVA indicated a reliable main effect of delay (*F*_4,516_ = 32.822, *MS*_*e*_ = 1.592 *p* < 0.001, ${\eta }_{p}^{2}$ = 0.203), and a reliable linear effect of delay (*F*_1,129_ = 67.756, *MS*_*e*_ = 2.970 *p* < 0.001, ${\eta }_{p}^{2}$ = 0.344) indicating that the participants showed a robust discounting effect.

Individual non-linear regressions were performed for each participant to derive the discount function (*k*) using Mazur’s (1987) hyperbolic discount function (1). Where the subjective discounted value (v) is estimated from the preferred smaller sooner outcome (*v*) for the five delay intervals (D). Because *k* is typically skewed it was transformed to their base-10 logarithms (log-*k*) for analyses.

Table 1 shows the average discount rates (log-*k*), BPVS scores, AQ scores, and SWAN scores for males and females. There were reliable differences between males and females in AQ and SWAN Scores, but not in discount rates or BPVS.

**Table 1:** Average discount rates (log-*k*), BPVS scores, AQ scores, and SWAN scores for males and females.

	Male			Female			*t*	*p*
	Mean	n	SD	Mean	n	SD		
Discount rate (*k*)	− 6.892	83	1.529	− 6.949	67	1.319	0.241	.810
**BPVS-3**								
Raw score	115.875	84	26.473	118.132	68	22.777	0.560	.576
Standardised score	105.134	82	13.707	102.250	67	11.828	1.364	.175
Equivalent year	8.536	82	2.885	8.537	67	2.245	.002	.999
AQ-Child	64.090	82	18.226	50.620	66	14.932	4.828	< .001
**SWAN**								
Inattention	− 0.318	82	0.967	− 0.829	66	0.808	3.436	< .001
Hyperactive/impulsive	− 0.446	82	0.983	− 0.819	66	0.973	2.310	< .022
Combined	− 0.382	82	0.849	− 0.825	66	0.785	3.261	< .001

The indices of multiple deprivation (IMD) were computed from the demographic information provided by parents using the publicly available resource provided by the Ministry of Housing, Communities and Local Government (http://imd-by-postcode.opendatacommunities.org/). This provides decile ranks from 1 = most deprived to 10 = least deprived. These were collapsed into quintiles (from 1 to 5). The average subjective discounted value for delay period for each IMD decile are shown in Table 2.

**Table 2:** Average age, subjective discounted value of £10 and discount rate (log-*k*) for each decile of multiple deprivation.

IMD		Age		Delay											
Quintile	n	Mean	SD	7-days		14-days		30-days		180-days		365-days		Discount rate (log-*k*)	
				Mean	SD	Mean	SD	Mean	SD	Mean	SD	Mean	SD	Mean	SD
1	18	8.356	2.060	8.778	1.457	8.187	2.337	7.559	2.963	7.464	2.257	6.786	3.173	− 6.111	1.748
2	19	8.243	1.978	9.289	0.384	8.868	0.704	8.789	1.018	7.289	2.893	6.917	3.469	− 6.755	1.561
3	31	8.723	2.100	8.661	1.881	8.283	2.156	8.500	1.402	7.481	2.199	6.552	2.971	− 6.634	1.565
4	33	8.567	2.113	9.109	1.517	8.906	0.902	8.742	1.040	7.968	2.105	8.000	2.017	− 7.234	1.133
5	53	8.118	2.079	9.260	0.394	8.706	1.001	8.570	1.355	8.410	1.466	8.240	1.523	− 7.269	1.081

**Figure 3:**
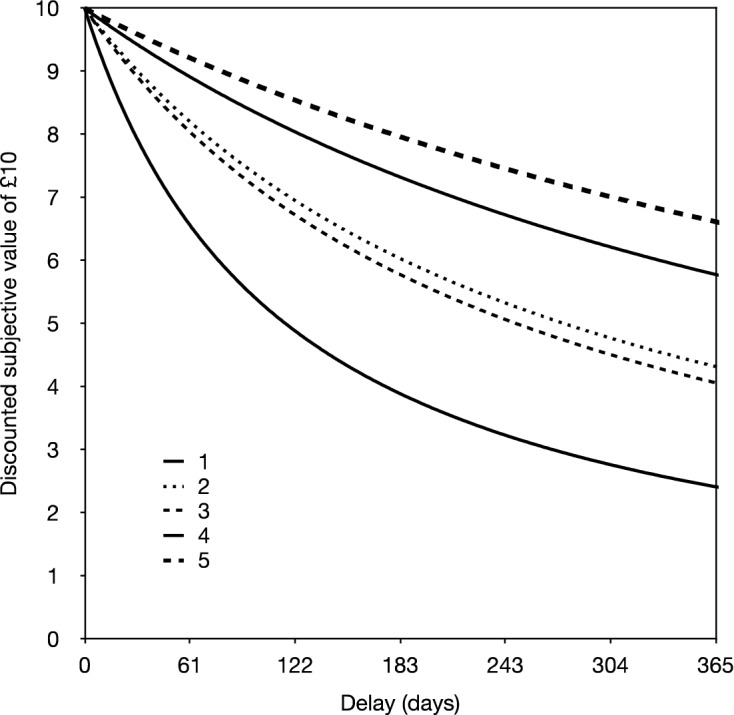
Showing the implied discount curves for each index of multiple deprivation quintile from 1 = most deprived to 5 = least deprived.

Figure 3 shows the implied discount curves for each IMD quintile. A one-way ANOVA on the log-transformed discount rates with IMD quintile as a between-subject factor was significant (*F*_1,149_ = 3.109, *MS*_*e*_ = 1.929, *p* = 0.017, ${\eta }_{p}^{2}$ = 0.080) indicating that there are reliable differences in impulsive choice made by children from different economic and social backgrounds. Planned contrasts showed that participants from the most deprived postcodes (IMD 1) show steeper discounting than participants in the least deprived postcodes (IMD 5, *p* < 0.003, and IMD 4, *p* = 0.007), but not the intermediate postcodes (IMD 3, *p* = 0.212; IMD 2, *p* = 0.161).

Finally, to test the hypothesis that relative deprivation is a casual factor in the development of impulsivity the IMD quintiles, age, gender, AQ score, SWANN score, and standardised BPVS-3 scores were entered into a linear regression as predictors of the log-transformed discount function. The model was significant (R^2^ = 0.190, *se* = 1.131, *F*_6,135_ = 5.037, *MS*_*e*_ = 1.718, *p* < 0.001). Table 3 shows the regression coefficients. Only age and the Index of Multiple Deprivation were reliable predictors of discount rates. Because younger children tend to discount more than older children it is possible that the effect of deprivation might be due to differences in the distribution of ages across the IMD quintiles. The average age of participants in each quintile are shown in Table 3 and suggest that this is not the case. A one-way ANOVA on age by quintile eliminated this possibility *F*_4,155_ < 1.0). Deprivation is a reliable and independent predictor of discount rates in children.

**Table 3:** Regression coefficients predictor variables onto discount rates.

	Regression coefficients		
	*β*	*t*	*p*
Age	0.294	3.600	0.001
Sex	0.061	0.684	0.495
Standardised BPVS-3	0.046	0.543	0.588
AQ-child	0.163	1.833	0.069
SWANN combined	0.099	1.172	0.243
IMD quintile	− 0.247	− 3.013	0.003

## Discussion

Relative deprivation predicts delay discounting in children aged 4 to 12 years. The data confirm a related report of a link between Indices of Multiple Deprivation and time preferences based on the English Longitudinal Study of Aging^29^. This adds to the growing evidence that economic uncertainty is an environmental factor in an individual difference in decision-making that is associated with a range of individual and social issues.

Previous research has shown a relationship between impulsive behaviours such as gambling and both social status based on occupation and relative deprivation based on IMD^35^. For example, unhealthy diets resulting in high BMI and obesity in both children^36^ and adults^37^. Both delay discounting^11,38^ and relative deprivation^39^ are key risk factors in the severity of tobacco dependence. Similarly, excessive alcohol consumption is also associated with relative deprivation^40^ and delay discounting^14^. One previous study explored if time preferences play a mediating role between relative deprivation and smoking and BMI^41^. Time preference in that study was measured using a psychometric scale Consideration of Future Consequences Scale (CFCS) that is only partially related discount rates^42^. Although this study did not find a clear relationship between time preferences and smoking that suggests that it is not as a robust measure of time preferences as discount rates. Nonetheless there is considerable evidence that discount rates as a stable individual difference may be a risk factor in for impulsive behaviours and poor health. The evidence presented here indicates that at least some of the individual difference in the impulsivity is associated with relative deprivation.

The relationship between childhood obesity and relative deprivation is particularly interesting when impulsivity is viewed through the lens of ecological models of foraging. Obesity in nursery age children is associated with both relative deprivation and food insecurity^43^. Evidence from the Bradford Cohort study shows that families living with relative deprivation tend to consume fewer fruit and vegetables and more high sugar drinks than more affluent families^44^. The UK Millennium Cohort study of 7262 children aged 11, showed that two thirds of children living in the most deprived areas in England were overweight or obese, compared to one fifth in the least deprived areas^45^. The link between food insecurity and relative deprivation is thought to be food consumption cycling in which food, and particularly energy dense food, is overconsumed in periods of abundance in anticipation of periods of scarcity^46,47^. This mechanism is synonymous if not identical to the Thrifty Phenotype Hypothesis^48^ and the Insurance Hypothesis^49^.

Early experience of relative deprivation, economic uncertainty, or food scarcity could result in a shift in choice parameters to generally prefer smaller sooner rather than larger later rewards. If this shift becomes stable, then it may result in overconsumption and unhealthy behaviours and a risk of developing dependencies and addictions throughout life^29^. Since 2010 the number of families in poverty in the United Kingdom has increased. Relative poverty in the UK has essentially unchanged in from 2007 to 2020 at around 22% of households. However, relative child poverty has continued to increase to around 700,000^50^. Between 2020 and 2021, the UKs largest network of food banks distributed 2.5 million emergency food parcels to people in crisis, a 33% increase the number of parcels and a 53% increase in the volume of parcels on the previous year^51^. Of these nearly 1 million went to children. Many of these were due to problems with the benefits system including delays, insufficient welfare payments and cuts to welfare payments. The Trussell Trust reported that in 2020 72% of families who received a food parcel had a family member with poor mental health. If it is the case that relative deprivation in childhood is causally related to impulsivity related health inequalities in adult life then current welfare policies may be creating future health inequalities and potentially a future public crisis in mental health.

## Limitations

This study used hypothetical rather than real monetary rewards. Real monetary rewards provide more compelling findings than hypothetical rewards in any decision-making task. However, this is challenging in delay discounting where the rewards could be delayed for months or years, and in children for whom the perception of monetary value or time might differ from adults. A recent systematic review of delay discounting in children reported 25 studies, of which only 8 involved real rewards, of which only 5 were monetary rewards^18^. Nonetheless there is a general consensus that delay discounting, even with hypothetical rewards, produces meaningful and reliable results. The cross-sectional nature of this study, both in terms of age and demographics, precludes any firm conclusions being drawn on the causal relationship between deprivation and impulsivity.

## Supplementary Information

Supplementary Table S1.

## Data Availability

All data generated or analysed during this study are included in this published article and its supplementary information file (Supplementary Table S1).
