# Supplementary material for: Economic and social deprivation predicts impulsive choice in children
Source: Sci Rep. 2022 May 27;12:8942. doi: 10.1038/s41598-022-12872-4 (PMC9142580; doi:10.1038/s41598-022-12872-4)
Supplement: Supplementary file 1 — Supplementary Table S1. [file 41598_2022_12872_MOESM1_ESM.docx]

Supplementary Table S1

Raw data used in the analyses.

| Age | Gender | IMD | BPVS | k | log-k | sex | IMD_Quintile | Seven_days | Fourteen_days | One_month | Six_months | One_year | AQ score | SWAN | BPVS raw | Language |
| --- | --- | --- | --- | --- | --- | --- | --- | --- | --- | --- | --- | --- | --- | --- | --- | --- |
| 10.43 | M | 9 | 114 | 0.00 | -8.55 | 1 | 5 | 9.50 | 9.50 | 9.50 | 9.50 | 9.50 | 90 | 1.17 | 150 | English |
| 6.57 | M | 8 | 100 | 0.00 | -8.37 | 1 | 4 | 1.00 | 9.50 |  | 9.50 | 9.50 | 58 | 0.17 | 97 | English |
| 7.84 | F | 8 | 102 | 0.01 | -4.97 | 0 | 4 | 9.50 | 9.50 | 8.50 | 2.00 | 5.50 | 53 | 0.06 | 108 | English |
| 8.89 | M | 10 | 127 | 0.01 | -5.11 | 1 | 5 | 9.50 | 6.50 | 6.50 | 5.00 | 5.00 | 75 | -0.67 | 149 | English |
| 8.45 | M | 10 | 122 | 0.00 | -7.17 | 1 | 5 | 9.50 | 9.50 | 9.00 | 9.50 | 7.50 | 70 | -0.28 | 133 | English |
| 8.81 | F | 3 | 99 | 0.00 | -5.60 | 0 | 2 | 8.50 | 7.50 | 9.50 | 1.00 | 9.50 | 47 | 0.56 | 120 | English |
| 9.54 | M | 2 | 83 | 0.00 | -5.39 | 1 | 1 | 9.50 | 9.50 |  |  |  | 76 | -2.00 | 113 | English and Urdu |
| 10.70 | F | 7 | 84 | 0.00 | -6.31 | 0 | 4 | 9.50 | 8.50 | 8.00 | 7.50 | 6.50 | 44 | -2.89 | 120 | English |
| 10.78 | F | 3 | 103 | 0.00 | -6.17 | 0 | 2 | 9.50 | 9.50 | 8.50 | 9.00 | 4.50 | 48 | 0.67 | 142 | English |
| 8.91 | M | 6 | 125 | 0.00 | -8.25 | 1 | 3 | 9.50 | 9.50 | 9.50 | 9.00 | 9.50 | 25 | -0.50 | 146 | English |
| 7.81 | M | 9 | 124 | 0.00 | -8.55 | 1 | 5 | 9.50 | 9.50 | 9.50 | 9.50 | 9.50 | 47 | 0.22 | 135 | English |
| 9.13 | F | 6 | 106 | 0.00 | -8.54 | 0 | 3 | 9.00 | 9.50 | 9.50 | 9.50 | 9.50 | 32 | -0.89 | 135 | English |
| 6.20 | M | 9 | 116 |  |  | 1 | 5 |  |  |  |  |  | 73 | -0.72 | 114 | English |
| 5.60 | F | 3 | 109 | 0.00 | -8.55 | 0 | 2 | 9.50 | 9.50 | 9.50 | 9.50 | 9.50 | 81 | -1.83 | 93 | English |
| 11.22 | F | 5 | 93 | 0.00 | -7.39 | 0 | 3 | 9.50 | 9.50 | 9.00 | 7.00 | 9.50 | 69 | 0.17 | 133 | English |
| 8.78 | F | 10 | 108 | 0.00 | -7.53 | 0 | 5 | 9.50 | 9.50 | 9.50 | 9.00 | 8.50 | 38 | -1.22 | 131 | English |
| 8.51 | M | 10 | 131 | 0.00 | -8.55 | 1 | 5 | 9.50 | 9.50 | 9.50 | 9.50 | 9.50 | 18 | -0.61 | 150 | English |
| 8.48 | F | 10 | 98 | 0.00 | -6.92 | 0 | 5 | 9.50 | 9.50 | 9.00 | 8.50 | 7.50 | 49 | 0.00 | 108 | English |
| 9.03 | M | 9 | 86 | 0.00 | -7.76 | 1 | 5 | 9.50 | 8.50 | 9.00 | 9.00 | 9.00 | 63 | -0.06 | 115 | English |
| 11.08 | F | 9 | 106 | 0.01 | -5.20 | 0 | 5 | 9.50 | 9.50 | 8.50 |  |  | 58 | -0.28 | 146 | English |
| 11.41 | F | 8 | 94 | 0.01 | -4.86 | 0 | 4 | 9.50 | 8.50 | 9.50 | 3.50 |  | 28 | -0.11 | 136 | English |
| 7.05 | M | 2 | 88 | 0.00 | -8.55 | 1 | 1 | 9.50 | 9.50 | 9.50 | 9.50 | 9.50 | 46 | -0.22 | 87 | English |
| 9.79 | M | 3 | 103 | 0.00 | -8.00 | 1 | 2 | 9.50 | 9.50 | 9.50 | 8.50 | 9.50 | 51 | 0.44 | 137 | English |
| 7.93 | M | 3 | 110 | 0.00 | -7.98 | 1 | 2 | 9.50 | 9.00 | 9.50 | 8.50 | 9.50 | 57 | 0.50 | 114 | English |
| 9.31 | M | 10 | 107 | 0.00 | -7.10 | 1 | 5 | 9.50 | 9.00 | 9.00 | 8.50 | 8.00 | 87 | -1.39 | 139 | English |
| 11.35 | M | 10 | 112 | 0.01 | -5.08 | 1 | 5 | 9.00 | 6.00 | 5.50 | 6.50 | 4.00 | 71 | -0.44 | 152 | English |
| 7.82 | M | 5 | 90 | 0.00 | -6.58 | 1 | 3 | 8.50 | 8.00 | 9.50 | 8.50 | 6.50 | 43 | -1.89 | 95 | English |
| 11.49 | M | 7 | 110 | 0.02 | -3.91 | 1 | 4 | 8.00 | 6.50 | 5.00 | 3.00 | 5.50 | 98 | -0.67 | 151 | English |
| 4.98 | F | 1 | 101 | 0.00 | -8.55 | 0 | 1 | 9.50 | 9.50 | 9.50 | 9.50 | 9.50 | 14 | 0.00 | 66 | English |
| 10.21 | M | 2 | 71 | 0.01 | -4.49 | 1 | 1 | 9.50 | 9.50 | 9.00 | 1.50 |  | 89 | 0.72 | 100 | English |
| 10.96 | F | 10 | 101 | 0.00 | -7.53 | 0 | 5 | 9.50 | 9.50 | 9.50 | 9.00 | 8.50 | 38 | -0.44 | 140 | English |
| 7.23 | M | 6 | 74 | 0.01 | -4.57 | 1 | 3 | 9.50 | 9.50 | 9.00 | 2.00 |  | 93 | 0.61 | 72 | English and Urdu |
| 7.11 | M | 2 | 97 |  |  | 1 | 1 | 6.00 | 0.50 | 0.50 | 9.00 | 0.50 | 58 | -1.94 | 97 | English |
| 9.29 | M | 6 | 83 | 0.00 | -7.77 | 1 | 3 | 9.00 | 9.00 | 9.00 | 9.00 | 9.00 | 101 | 0.33 | 113 | English and Urdu |
| 6.78 | F | 2 | 103 | 0.00 | -5.54 | 0 | 1 | 9.00 | 9.00 | 8.00 | 6.50 | 4.00 | 62 | -0.83 | 102 | English |
| 9.43 | F | 7 | 111 | 0.00 | -5.63 | 0 | 4 | 9.50 | 5.50 | 9.50 | 6.50 | 4.50 | 41 | -0.28 | 143 | English |
| 6.34 | M | 5 | 109 | 0.00 | -8.25 | 1 | 3 | 9.50 | 9.50 | 9.50 | 9.00 | 9.50 | 78 | -2.28 | 105 | English |
| 10.46 | M | 2 | 103 | 0.00 | -7.72 | 1 | 1 |  |  | 9.50 | 9.50 | 8.50 | 68 | -2.17 | 139 | English |
| 8.43 | M | 10 | 122 |  |  | 1 | 5 |  |  |  |  |  | 68 | 0.06 | 133 | English |
| 4.99 | F | 10 | 119 | 0.00 | -8.05 | 0 | 5 |  | 9.50 | 9.50 | 9.50 | 9.00 | 57 | -0.50 | 91 | English |
| 8.63 | F | 10 | 92 | 0.00 | -7.45 | 0 | 5 | 9.50 | 9.50 | 9.00 | 8.00 | 9.00 | 45 | -0.83 | 106 | English |
| 11.36 | M | 4 | 107 | 0.01 | -4.46 | 1 | 2 | 9.50 | 8.00 | 8.00 | 4.00 | 0.50 | 96 | 0.06 | 148 | English |
| 6.02 | F | 6 | 113 | 0.01 | -5.29 | 0 | 3 | 9.50 | 9.00 | 8.00 | 7.50 | 1.50 | 57 | -1.33 | 106 | English |
| 4.15 | F | 6 | 82 | 0.00 | -8.44 | 0 | 3 | 9.50 | 9.50 | 8.50 | 9.50 | 9.50 | 24 | -0.17 | 30 | English |
| 8.65 | F | 10 | 83 | 0.00 | -6.82 | 0 | 5 | 9.50 | 8.50 | 3.00 | 9.50 | 7.50 | 51 | -0.94 | 98 | English |
| 7.75 | M | 4 | 121 | 0.00 | -7.96 | 1 | 2 | 9.50 | 9.50 | 9.00 | 8.50 | 9.50 | 61 | 0.44 | 130 |  |
| 7.10 | M | 10 | 109 | 0.00 | -8.12 | 1 | 5 | 9.00 | 9.50 | 5.50 | 9.50 | 9.50 | 75 | -0.61 | 112 | English |
| 6.01 | F | 10 | 113 | 0.00 | -6.82 | 0 | 5 | 9.00 | 8.00 | 8.00 | 5.50 | 9.50 | 55 | -1.17 | 107 | English |
| 11.67 | M | 10 | 114 | 0.00 | -6.76 | 1 | 5 | 9.50 | 9.50 | 8.00 | 8.00 | 7.50 | 60 | -2.06 | 154 | English |
| 7.78 | F | 5 | 109 | 0.03 | -3.65 | 0 | 3 | 8.50 | 3.50 | 6.50 | 4.50 | 2.50 | 43 | -1.56 | 116 | English |
| 5.66 | F | 8 | 106 | 0.00 | -8.44 | 0 | 4 | 9.50 | 8.50 | 9.00 | 9.50 | 9.50 | 34 | -2.61 | 89 | English |
| 5.69 | M | 10 | 123 | 0.00 | -8.24 | 1 | 5 | 9.00 | 9.50 | 9.50 | 9.00 | 9.50 | 84 | 0.11 | 115 | English |
| 11.17 | F | 10 | 105 | 0.01 | -5.09 | 0 | 5 | 8.50 | 6.00 | 6.00 | 5.50 | 5.00 |  |  | 145 | English |
| 7.56 | M | 10 | 99 | 0.00 | -7.17 | 1 | 5 | 9.00 | 8.50 | 9.00 | 8.00 | 8.50 |  |  | 104 | English |
| 9.76 | M | 10 | 108 | 0.00 | -7.40 | 1 | 5 | 9.00 | 9.00 | 9.50 | 9.50 | 8.00 |  |  | 143 | English |
| 10.96 | M |  | 90 | 0.01 | -4.69 | 1 |  | 8.50 | 7.50 | 6.00 | 5.00 | 3.50 | 47 | -0.39 | 129 | English |
| 5.00 | M | 7 | 83 | 0.00 | -7.98 | 1 | 4 | 9.50 | 9.00 | 9.50 | 8.50 | 9.50 | 59 | 0.00 | 49 | Arabic |
| 6.70 | F | 7 |  |  |  | 0 | 4 |  |  |  |  |  | 55 | -0.78 |  | English |
| 8.48 | M | 7 |  | 0.00 | -7.69 | 1 | 4 | 9.50 | 9.50 | 9.50 | 9.50 | 8.50 | 65 | -0.33 | 69 | Arabic |
| 8.91 | M | 8 | 112 | 0.00 | -5.90 | 1 | 4 | 8.50 | 8.00 | 7.50 | 6.50 | 6.00 | 72 | -2.11 | 133 | English |
| 10.80 | M | 8 | 114 | 0.00 | -6.11 | 1 | 4 | 9.50 | 8.50 | 9.00 | 7.50 | 5.50 | 59 | -0.17 | 152 | English |
| 10.37 | F | 9 | 94 | 0.00 | -8.13 | 0 | 5 | 9.00 | 8.00 | 9.00 | 9.00 | 9.50 | 51 | -1.67 | 129 | English |
| 8.05 | M | 10 | 121 | 0.01 | -4.49 | 1 | 5 | 8.50 | 7.50 | 6.50 | 5.00 |  | 105 | 0.72 | 131 | English |
| 4.22 | M | 7 | 116 | 0.00 | -8.21 | 1 | 4 | 9.50 | 9.00 | 6.50 | 9.50 | 9.50 | 44 | -0.11 | 72 | English |
| 8.62 | M | 8 | 119 | 0.00 | -7.59 | 1 | 4 | 9.00 | 8.00 | 9.50 | 8.50 | 9.00 | 73 | -0.33 | 133 | English |
| 6.80 | M | 1 | 99 | 0.01 | -4.38 | 1 | 1 | 9.00 | 9.00 | 8.00 |  | 0.50 | 68 | -0.28 | 98 | English |
| 10.16 | M | 4 | 104 | 0.02 | -4.05 | 1 | 2 | 8.50 | 7.50 | 6.00 | 4.00 | 1.00 |  |  | 139 | English |
| 6.88 | M | 5 | 112 | 0.00 | -5.35 | 1 | 3 | 9.50 | 9.50 | 9.00 | 4.50 | 4.50 | 47 | -0.06 | 113 | English |
| 9.32 | F | 6 | 114 | 0.00 | -7.65 | 0 | 3 | 9.50 | 9.50 | 9.50 | 8.50 | 9.00 | 73 | -1.22 | 145 | English |
| 8.79 | F | 7 | 116 | 0.00 | -6.14 | 0 | 4 | 9.50 | 9.00 | 8.50 | 6.50 | 6.50 | 21 | -2.44 | 137 | English |
| 5.33 | F | 9 | 111 | 0.00 | -7.34 | 0 | 5 | 9.00 | 7.00 | 9.50 | 7.00 | 9.50 | 57 | -0.67 | 92 | English |
| 10.37 | F | 1 | 109 | 0.00 | -7.48 | 0 | 1 | 9.50 | 9.00 | 8.00 | 7.50 | 9.50 | 53 | -0.11 | 146 | English |
| 9.23 | M | 6 | 86 | 0.00 | -5.80 | 1 | 3 | 9.00 | 9.50 | 8.50 | 6.50 | 5.00 |  |  | 115 | English |
| 5.62 | M | 4 | 102 | 0.00 | -5.49 | 1 | 2 | 8.50 | 9.50 | 9.50 | 9.50 | 0.00 | 75 | -1.00 | 85 | English |
| 5.98 | F | 10 | 100 | 0.00 | -8.47 | 0 | 5 | 9.50 | 8.00 | 9.50 | 9.50 | 9.50 | 33 | -0.33 | 87 | English |
| 8.65 | F | 10 | 83 | 0.00 | -8.52 | 0 | 5 | 9.50 | 9.00 | 9.50 | 9.50 | 9.50 | 51 | -0.94 | 98 | English |
| 11.04 | M | 7 | 114 | 0.00 | -6.46 | 1 | 4 | 9.50 | 9.50 | 9.50 | 7.00 | 7.00 | 35 | -0.78 | 153 | English |
| 10.71 | F | 8 | 106 | 0.00 | -5.33 | 0 | 4 | 9.50 | 9.00 | 8.50 | 7.50 | 1.50 | 36 | -0.11 | 143 | English |
| 6.81 | F | 8 | 103 | 0.00 | -7.36 | 0 | 4 | 9.00 | 9.50 | 8.50 | 9.50 | 8.00 | 41 | -0.89 | 102 | English |
| 11.73 | F | 6 | 86 | 0.00 | -6.11 | 0 | 3 | 9.50 | 9.50 | 8.50 | 7.50 | 5.50 |  |  | 129 | English |
| 7.55 | F | 6 | 114 | 0.00 | -8.55 | 0 | 3 | 9.50 | 9.50 | 9.50 | 9.50 | 9.50 | 33 | -1.61 | 121 | English |
| 9.26 | F | 6 | 103 | 0.00 | -6.33 | 0 | 3 | 9.00 | 9.50 | 8.00 | 7.50 | 6.50 | 52 | -1.17 | 135 | English |
| 8.14 | F | 8 | 102 | 0.00 | -8.33 | 0 | 4 | 9.50 | 9.50 | 7.50 | 9.50 | 9.50 | 64 | -1.72 | 110 | English |
| 9.86 | M | 6 | 75 | 0.01 | -4.87 | 1 | 3 | 9.50 | 8.00 | 9.50 |  | 2.00 | 70 | 1.28 | 103 | English |
| 7.25 | M | 6 | 107 | 0.00 | -7.42 | 1 | 3 | 9.50 | 9.50 | 9.50 | 9.50 | 8.00 | 36 | -1.28 | 110 | English |
| 10.71 | M | 9 | 113 | 0.00 | -6.95 | 1 | 5 | 9.50 | 9.50 | 8.50 | 9.50 | 7.00 | 58 | -0.22 | 150 | English |
| 6.67 | M | 9 | 105 | 0.00 | -7.07 | 1 | 5 | 8.00 | 8.50 | 9.00 | 8.50 | 8.00 | 43 | 0.00 | 102 | English |
| 7.83 | F | 4 | 107 | 0.02 | -4.04 | 0 | 2 | 9.50 | 8.50 | 7.50 | 0.50 |  | 53 | -1.06 | 113 | English |
| 11.98 | F | 6 | 85 |  |  | 0 | 3 | 2.00 | 3.50 | 4.00 | 4.00 | 4.00 | 53 | -0.72 | 130 | French and English |
| 8.86 | M | 7 | 98 | 0.00 | -7.31 | 1 | 4 | 9.50 | 8.50 | 9.00 | 8.50 | 8.50 | 66 | 0.22 | 119 | English |
| 11.78 | M | 3 | 100 | 0.00 | -6.00 | 1 | 2 | 9.50 | 8.50 | 9.00 | 7.50 | 5.00 | 56 | -1.44 | 143 | English |
| 7.16 | F | 2 | 102 | 0.00 | -6.69 | 0 | 1 | 9.50 | 8.50 | 9.50 | 7.50 | 7.50 | 86 | 0.61 | 103 | English |
| 5.73 | F | 10 | 104 | 0.00 | -7.60 | 0 | 5 | 9.50 | 9.00 | 9.00 | 8.50 | 9.00 | 55 | -0.72 | 87 | English |
| 8.67 | M | 2 | 100 | 0.07 | -2.60 | 1 | 1 | 8.50 | 5.00 | 1.00 |  |  | 85 | 0.78 | 115 | English |
| 5.87 | M | 3 | 109 | 0.00 | -8.03 | 1 | 2 | 9.50 | 9.00 | 9.50 | 9.50 | 9.00 | 66 | 0.44 | 99 | English |
| 8.64 | F | 3 | 117 | 0.00 | -7.31 | 0 | 2 | 9.50 | 8.50 | 9.00 | 8.50 | 8.50 | 57 | -0.67 | 130 | English |
| 7.45 | F | 9 | 80 | 0.00 | -7.75 | 0 | 5 | 9.50 | 8.50 | 9.50 | 8.00 | 9.50 | 68 | -1.06 | 80 | English |
| 9.01 | M | 10 | 85 | 0.01 | -5.23 | 1 | 5 | 9.00 | 9.50 | 7.00 | 4.00 | 5.50 | 93 | 1.39 | 114 | English |
| 4.60 | M | 9 | 122 | 0.00 | -8.48 | 1 | 5 | 9.00 | 9.50 | 9.00 | 9.50 | 9.50 | 54 | 0.00 | 88 | English |
| 5.19 | M | 10 | 114 | 0.00 | -8.25 | 1 | 5 | 9.50 | 9.50 | 9.50 | 9.00 | 9.50 | 45 | 0.22 | 90 | English |
| 9.44 | F | 10 | 104 | 0.00 | -5.44 | 0 | 5 | 8.00 | 7.00 | 7.00 | 6.50 | 4.50 | 34 | -0.78 | 137 | English |
| 7.33 | M | 7 | 98 | 0.00 | -8.55 | 1 | 4 | 9.50 | 9.50 | 9.50 | 9.50 | 9.50 | 47 | -1.11 | 100 | Mandarin |
| 11.36 | F | 2 | 87 | 0.00 | -5.48 | 0 | 1 | 9.50 | 9.00 | 8.50 | 5.00 | 5.00 | 56 | -0.89 | 129 | English |
| 6.32 | M | 9 | 90 | 0.00 | -8.50 | 1 | 5 | 9.50 | 8.50 | 9.50 | 9.50 | 9.50 | 67 | 0.00 | 82 | English |
| 9.57 | M | 7 | 99 | 0.00 | -8.55 | 1 | 4 | 9.50 | 9.50 | 9.50 | 9.50 | 9.50 | 93 | -0.89 | 125 | Mandarin |
| 6.11 | M | 10 | 109 | 0.00 | -7.55 | 1 | 5 | 8.50 | 6.50 | 9.00 | 9.50 | 8.50 | 76 | -0.11 | 103 | English |
| 5.26 | M | 6 | 87 | 0.00 | -8.47 | 1 | 3 | 5.50 |  | 9.50 | 9.50 | 9.50 | 72 | -1.50 | 62 | Japanese and German |
| 5.85 | M | 9 | 114 | 0.00 | -6.69 | 1 | 5 | 9.50 | 8.50 | 9.50 | 7.50 | 7.50 | 58 | -0.50 | 107 | English |
| 9.36 | F | 4 | 114 | 0.00 | -6.36 | 0 | 2 | 9.00 | 9.00 | 7.50 | 6.50 | 7.50 | 42 | -1.28 | 145 | English |
| 10.56 | M | 6 | 91 | 0.01 | -4.81 | 1 | 3 | 9.50 | 9.00 | 7.50 | 4.00 | 3.00 | 63 | -2.06 | 128 | English |
| 6.11 | M | 4 |  | 0.00 | -8.55 | 1 | 2 | 9.50 | 9.50 | 9.50 | 9.50 | 9.50 | 34 | -0.89 |  | English |
| 9.00 | F | 2 | 84 | 0.00 | -6.51 | 0 | 1 | 8.50 |  | 3.50 | 6.00 | 9.00 | 91 | -0.17 | 113 | English |
| 8.30 | M | 7 | 123 | 0.00 | -8.03 | 1 | 4 | 9.50 | 9.00 | 9.50 | 9.50 | 9.00 | 35 | -0.22 | 136 | English |
| 8.20 | F | 4 | 122 | 0.00 | -8.55 | 0 | 2 | 9.50 | 9.50 | 9.50 | 9.50 | 9.50 | 49 | -0.56 | 132 | English |
| 11.65 | M | 7 | 89 | 0.00 | -8.55 | 1 | 4 | 9.50 | 9.50 | 9.50 | 9.50 | 9.50 | 25 | -0.61 | 133 | English |
| 9.52 | F | 6 | 91 | 0.00 | -8.55 | 0 | 3 | 9.50 | 9.50 | 9.50 | 9.50 | 9.50 | 48 | -1.89 | 124 | English |
| 11.52 | M | 9 | 112 | 0.00 | -6.43 | 1 | 5 | 9.50 | 9.50 | 9.00 | 9.00 | 5.50 | 61 | -1.11 | 152 | English |
| 7.55 | F | 3 | 108 | 0.00 | -5.58 | 0 | 2 | 9.50 | 9.50 | 9.50 | 6.00 | 4.00 | 35 | -1.50 | 113 | English |
| 8.93 | M | 5 | 116 |  |  | 1 | 3 | 2.50 | 1.00 |  |  |  | 72 | -1.06 | 137 | English |
| 8.22 | M | 3 | 110 | 0.00 | -7.64 | 1 | 2 | 9.50 | 8.00 | 7.50 | 9.00 | 9.00 | 63 | -0.39 | 117 | English |
| 8.21 | F | 7 | 114 | 0.00 | -8.55 | 0 | 4 | 9.50 | 9.50 | 9.50 | 9.50 | 9.50 | 56 | -0.44 | 121 | English |
| 11.05 | F | 9 | 105 | 0.00 | -7.12 | 0 | 5 | 9.50 | 9.50 | 9.00 | 8.50 | 8.00 | 69 | -0.89 | 140 | English |
| 10.54 | F | 7 | 108 | 0.00 | -8.49 | 0 | 4 | 9.50 | 9.50 | 9.00 | 9.50 | 9.50 | 58 | -1.56 | 146 | English |
| 6.59 | M | 2 | 88 | 0.00 | -8.44 | 1 | 1 | 9.50 | 7.50 | 9.50 | 9.50 | 9.50 | 78 | -0.72 | 82 | English |
| 9.18 | F | 2 | 91 | 0.00 | -7.08 | 0 | 1 | 9.50 | 9.00 | 9.50 | 7.50 | 8.50 | 75 | 1.67 | 119 | English |
| 10.67 | F | 5 | 86 | 0.00 | -6.38 | 0 | 3 | 9.50 | 9.50 | 5.50 |  | 7.00 | 50 | -1.06 | 123 | English |
| 7.71 | M | 6 | 100 | 0.00 | -8.31 | 1 | 3 | 8.00 | 5.50 | 9.50 | 9.50 | 9.50 | 59 | -0.56 | 105 | English |
| 12.13 | M | 6 | 115 | 0.00 | -6.11 | 1 | 3 | 9.00 | 8.50 | 9.50 | 8.00 | 5.00 | 55 | -1.50 | 155 | English |
| 9.00 | M | 10 |  | 0.00 | -8.14 | 1 | 5 | 9.50 | 9.00 | 8.50 | 9.00 | 9.50 | 61 | 1.11 |  | English |
| 5.25 | M | 4 | 102 | 0.00 | -8.02 | 1 | 2 | 9.00 | 9.00 | 9.50 | 9.50 | 9.00 | 36 | 0.00 | 79 | English |
| 6.81 | M | 7 | 103 | 0.00 | -8.03 | 1 | 4 | 9.00 | 9.50 | 9.50 | 9.50 | 9.00 | 77 | 0.44 | 102 | English |
| 4.23 | F | 7 | 125 | 0.00 | -8.55 | 0 | 4 | 9.50 | 9.50 | 9.50 | 9.50 | 9.50 | 45 | -1.11 | 86 | English |
| 10.32 | F | 7 | 100 | 0.00 | -7.50 | 0 | 4 | 9.50 | 9.50 | 9.00 | 9.00 | 8.50 | 60 | -1.83 | 135 | English and Arabic |
| 5.03 | M | 2 | 96 | 0.02 | -3.85 | 1 | 1 | 4.00 | 9.50 | 7.00 |  |  | 56 | 0.11 | 68 | English |
| 8.61 | M | 10 |  | 0.00 | -5.75 | 1 | 5 | 9.50 | 7.50 | 8.50 | 5.50 | 6.00 | 91 | -1.00 |  | English |
| 4.70 | M | 5 | 115 | 0.00 | -7.82 | 1 | 3 | 9.50 | 9.00 | 9.50 | 9.00 | 9.00 | 53 | 0.00 | 78 | English |
| 12.06 | M | 2 | 91 | 0.00 | -6.24 | 1 | 1 | 9.50 | 8.50 | 8.50 | 7.00 | 6.50 | 54 | -0.39 | 137 | English |
| 10.14 | F | 6 | 86 | 0.00 | -7.21 | 0 | 3 | 9.50 | 9.00 | 8.50 | 9.00 | 8.00 | 71 | -0.17 | 125 | English |
| 5.32 | M | 10 | 118 | 0.00 | -6.98 | 1 | 5 | 9.50 | 9.50 | 8.00 | 9.00 |  | 78 | -0.72 | 105 | English |
| 4.30 | F | 10 | 116 | 0.00 | -7.65 | 0 | 5 | 9.50 | 9.50 | 9.50 | 8.50 | 9.00 | 49 | -1.00 | 74 | English |
| 8.37 | F | 10 | 117 | 0.00 | -7.53 | 0 | 5 | 9.50 | 9.50 | 9.50 | 9.00 | 8.50 | 52 | -0.78 | 126 | English |
| 8.93 | F | 6 | 116 | 0.02 | -3.81 | 0 | 3 | 9.00 | 8.00 |  |  | 0.00 | 29 | -0.89 | 137 | English |
| 9.26 | F | 9 | 101 | 0.00 | -8.21 | 0 | 5 | 9.00 | 8.00 |  | 9.00 | 9.50 | 39 | -0.72 | 130 | English |
| 11.21 | F | 7 | 80 | 0.00 | -8.25 | 0 | 4 | 9.50 | 9.50 | 9.50 | 9.00 | 9.50 | 64 | -1.94 | 118 | English and Arabic |
| 7.85 | F | 8 | 108 | 0.00 | -6.72 | 0 | 4 | 9.50 | 9.00 | 8.00 | 5.00 | 9.50 | 60 | -0.33 | 114 | English |
| 6.70 | M | 7 | 113 | 0.00 | -8.50 | 1 | 4 | 9.50 | 9.00 | 8.50 |  | 9.50 | 72 | -0.44 | 112 | English |
| 9.81 | F | 7 | 89 | 0.00 | -6.30 | 0 | 4 | 9.50 | 9.00 | 8.00 | 8.00 | 6.00 | 57 | -0.89 | 123 | English |
| 9.39 | M | 5 | 94 | 0.01 | -4.67 | 1 | 3 | 8.50 | 6.50 | 6.50 | 5.00 | 3.50 |  |  | 124 | English |
| 8.11 | M | 10 | 126 | 0.00 | -7.98 | 1 | 5 | 9.50 | 9.00 | 9.00 | 9.50 | 9.00 | 64 | -1.06 | 140 | English |
| 6.96 | M | 10 | 98 | 0.00 | -7.95 | 1 | 5 | 9.50 | 9.00 | 8.50 | 9.50 | 9.00 | 78 | 0.33 | 96 | English |
| 6.23 | M | 10 |  | 0.00 | -7.68 | 1 | 5 | 9.50 | 9.00 | 9.50 | 9.50 | 8.50 | 51 | 0.28 | 46 |  |
| 11.52 | F | 5 | 85 | 0.00 | -5.44 | 0 | 3 | 9.50 | 8.50 | 7.00 | 5.50 | 5.00 |  |  | 128 | English |
| 11.65 | F | 10 | 117 | 0.00 | -7.33 | 0 | 5 | 9.00 | 8.50 | 8.50 | 9.50 | 8.00 | 35 | -1.39 | 156 | English |
| 9.61 | M | 2 | 110 | 0.00 | -6.86 | 1 | 1 | 9.00 | 8.50 | 9.50 | 9.00 | 7.00 | 58 | -0.72 | 144 | English |
| 5.45 | F | 10 | 123 | 0.00 | -8.52 | 0 | 5 | 9.50 | 9.00 | 9.50 | 9.50 | 9.50 | 57 | -0.33 | 111 | English |
| 6.81 | M | 2 | 126 | 0.02 | -4.14 | 1 | 1 | 9.00 |  |  |  |  | 68 | 1.06 | 131 | English |
